# Adhesive Hydrogel Building Blocks to Reconstruct Complex Cartilage Tissues

**DOI:** 10.1021/acsbiomaterials.2c01438

**Published:** 2023-03-07

**Authors:** Connor J. Demott, McKenzie R. Jones, Caleb D. Chesney, Melissa A. Grunlan

**Affiliations:** † Department of Biomedical Engineering, Texas A&M University, College Station, Texas 77843-3003, United States; ‡ Department of Materials Science & Engineering, Texas A&M University, College Station, Texas 77843-3003, United States; § Department of Chemistry, Texas A&M University, College Station, Texas 77843-3003, United States

**Keywords:** triple network hydrogel, adhesion, surface, cartilage, electrostatic

## Abstract

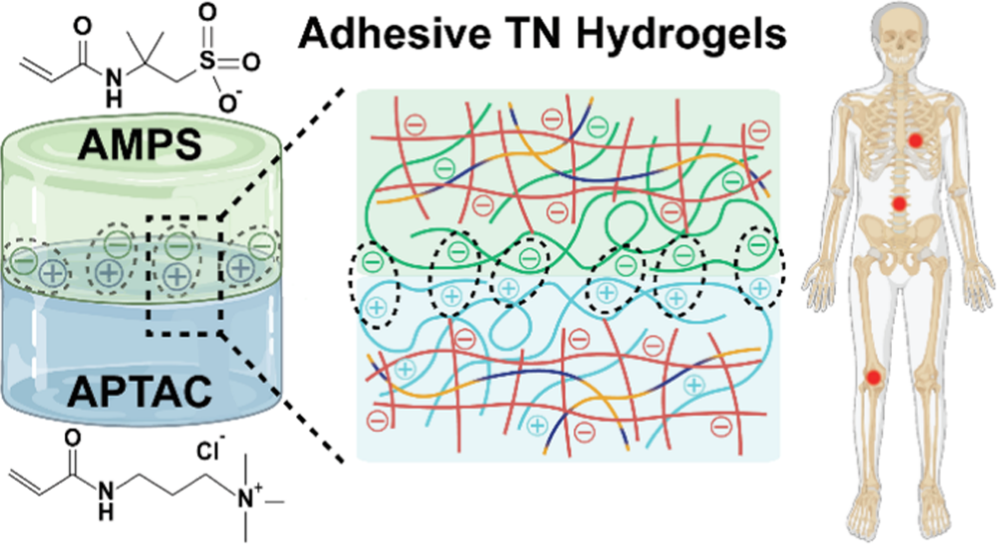

Cartilage has an intrinsically low healing capacity, thereby requiring surgical intervention. However, limitations of biological grafting and existing synthetic replacements have prompted the need to produce cartilage-mimetic substitutes. Cartilage tissues perform critical functions that include load bearing and weight distribution, as well as articulation. These are characterized by a range of high moduli (≥1 MPa) as well as high hydration (60–80%). Additionally, cartilage tissues display spatial heterogeneity, resulting in regional differences in stiffness that are paramount to biomechanical performance. Thus, cartilage substitutes would ideally recapitulate both local and regional properties. Toward this goal, triple network (TN) hydrogels were prepared with cartilage-like hydration and moduli as well as adhesivity to one another. TNs were formed with either an anionic or cationic 3^rd^ network, resulting in adhesion upon contact due to electrostatic attractive forces. With the increased concentration of the 3^rd^ network, robust adhesivity was achieved as characterized by shear strengths of ∼80 kPa. The utility of TN hydrogels to form cartilage-like constructs was exemplified in the case of an intervertebral disc (IVD) having two discrete but connected zones. Overall, these adhesive TN hydrogels represent a potential strategy to prepare cartilage substitutes with native-like regional properties.

## Introduction

**Figure 1:**
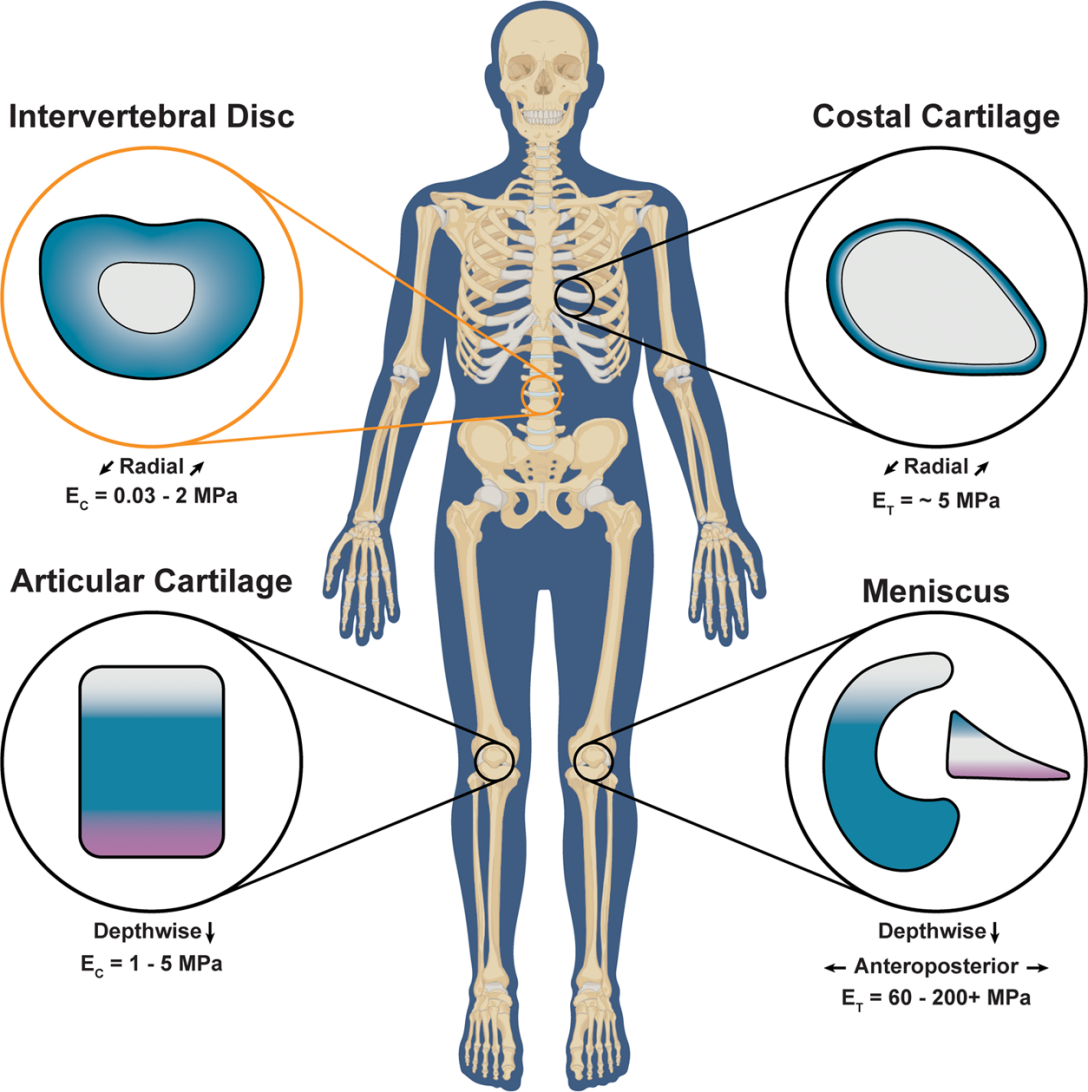
Cartilage tissues throughout the body exhibit regional properties (e.g., depthwise and radial). These regional properties give rise to unique mechanical functions to support articulation and load bearing.^3,5−14^ Representative IVD-like construct illustrated in this work (orange). *E*_C_ = compressive modulus and *E*_T_ = tensile modulus.

Cartilaginous tissues perform critical roles in load bearing and distribution, support, and motion throughout the body.^1−4^ Distinct biomechanical properties are associated with regional differences found in most cartilage tissues (e.g., articular cartilage, meniscus, costal cartilage, intervertebral discs (IVDs); Figure 1).^3,5−13^ For instance, IVDs have two major regions: the annulus fibrosus (AF) and the nucleus pulposus (NP). The NP is a gelatinous core, while the AF is a rigid, fibrocartilage ring composed of concentric lamellae. This unique combination allows for IVDs to resist compression yet still allow for flexion/extension, bending, and rotation.^6,14^ When damaged or degenerated, clinical repair of cartilage is often limited due to avascularity and structural alterations.^14−18^ Biological grafting is most often leveraged, as well as other surgical procedures such as microfracture for articular cartilage.^15,19−21^ Nevertheless, such procedures remain constrained by graft availability, donor site morbidity, and fibrocartilage formation.^18,19,22−25^ For severe cartilage degeneration, additional instrumentation may be required that sacrifices native biomechanics and can lead to damage to adjacent tissues (e.g., spinal fusion).^26−29^ Artificial replacements have thus emerged, commonly combining metallic and hard polymeric materials to withstand the high load-bearing environment (e.g., artificial IVDs or articular cartilage focal resurfacing devices).^30−33^ However, these fail to properly replicate tissue mechanics. Specifically, these devices suffer from a mechanical mismatch with surrounding cartilage tissue, leading to degeneration and poor integration.^14,34−38^ This can be partially attributed to their lack of hydration, as osmotic forces of hydrated (60–90% water) cartilage tissues dictate their mechanics (e.g., moduli and viscoelasticity).^39−41^ While hydrogels can be prepared with high hydration, most hydrogels exhibit compressive moduli that are orders of magnitude lower than most cartilage tissues and further lack characteristic regional differences.^2,3,6,42,43^ Thus, there is a need for hydrogel cartilage substitutes that are more cartilage mimetic.

Multilayered hydrogels have been fabricated using various in situ, multistep processes.^44,45^ To mimic the depth-dependent properties of articular cartilage, Nguyen et al. developed a trilayer poly(ethylene glycol) (PEG)-based construct.^46^ This was accomplished via a sequential photopolymerization process wherein each layer’s precursor solution was cured on top of a partially cured hydrogel layer to permit a thin “mixed” region between layers. However, the moduli of each layer did not parallel that of the native cartilage tissue layers. Regional properties may also be afforded using adhesive hydrogels. Adhesive hydrogels have been reported based on polyelectrolytes (PEs) and polyampholytes (PAs). PEs are based on anionic or cationic monomers, while PAs comprise monomers of a balance of opposite charges (i.e., 50:50—positive/negative).^47,48^ For PE and PA hydrogels, adhesivity is achieved via ionic bonding to charged surfaces.^47^ PE hydrogels achieve adhesion via electrostatic interactions with oppositely charged surfaces. In the case of PA hydrogels, “self-adjustable” adhesion can be achieved (i.e., to either cationic or anionic surfaces) as well as to tissue.^48,49^ Nonetheless, PE and PA hydrogels having cartilage-mimetic moduli have not been reported.

Multinetwork hydrogels offer a strategy to achieve robust mechanical properties.^50−52^ Recently, our group reported triple network (TN) hydrogels that leveraged both electrostatic and hydrophobic interactions to achieve unprecedented, cartilage-matching moduli (∼1 to ∼3 MPa) and hydration (∼80%).^53^ The synergy and dynamic nature of these physical cross-links also afforded high strength and toughness. These were composed of asymmetrically cross-linked networks of anionic poly(2-acrylamido-2-methylpropane sulfonic acid) (PAMPS) and poly(*N*-isopropylacrylamide-*co*-acrylamide) (P(NIPAAm-*co*-AAm)) and cationic poly((3-acrylamidopropyl)trimethylammonium chloride) (PAPTAC). The NIPAAm units of the 2^nd^ network provided hydrophobic interactions. To ensure dimensional stability (i.e., no swelling/deswelling) under physiologic conditions, the volume phase transition temperature (VPTT) was tuned beyond the physiologic range through the copolymerization of AAm in the 2^nd^ network.^51,53^ Despite their cartilage-like hydration and moduli, these TN-APTAC hydrogels do not mimic the regional properties exhibited by most cartilaginous tissues.

**Figure 2:**
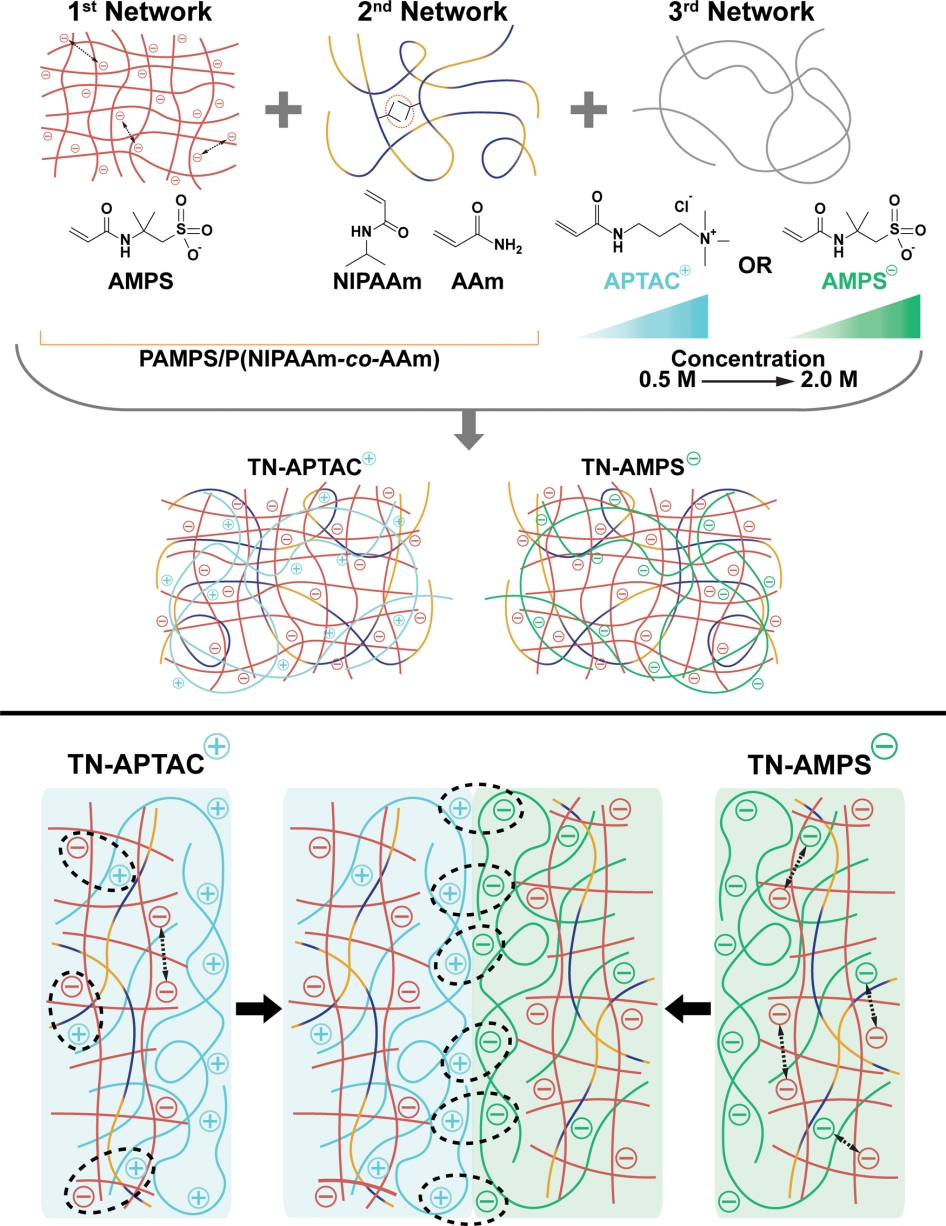
Top: TN hydrogels were fabricated with either a cationic (TN-APTAC) or anionic (TN-AMPS) 3^rd^ network, wherein the concentration of APTAC or AMPS was tuned (0.5–2.0 M). Bottom: the 3^rd^ network in TN hydrogels drives surface charge, enabling adhesion between the two types via electrostatic attractive forces.

Herein, toward preparing cartilage-mimetic hydrogel constructs with regional properties, we sought to demonstrate the adhesivity of TN hydrogels imparted by oppositely charged 3^rd^ networks (Figure 2). It has been reported that the final network of multinetwork hydrogels drives surface properties.^54,55^ Thus, TN hydrogels were prepared with cationic (TN-APTAC^53^) or anionic 3^rd^ networks (TN-AMPS) of varying concentrations (0.5–2.0 M) to afford their adhesion to one another. For both TN types, the 1^st^ network was composed of tightly cross-linked and anionic PAMPS, and the 2^nd^ network was loosely cross-linked P(NIPAAm-*co*-AAm). The resulting TN hydrogel types varied not only in terms of surface charge but also intra- and internetwork interactions within the bulk. TN-APTAC hydrogels afforded electrostatic attractive interactions between the anionic 1^st^ and cationic 3^rd^ networks. In contrast, TN-AMPS hydrogels produced electrostatic repulsive interactions between the mutually anionic 1^st^ and 3^rd^ networks. Characterization of hydration and mechanical properties was completed, with moduli assessed under low stains relevant to physiological loading. Adhesion between the cationic TN-APTAC and anionic TN-AMPS hydrogels was evaluated through lap shear testing. Finally, the ability of oppositely charged TN hydrogels to be used in the development of heterogeneous cartilage replacements was analyzed with the development of a proof-of-concept artificial IVD-like construct, and a design for articular cartilage was proposed.

## Experimental Section

### Materials

Acrylamide (AAm, >99%), 2-acrylamido-2-methylpropane sulfonic acid (AMPS, 97%), (3-acrylamidopropyl)trimethylammonium chloride solution (APTAC, 75 wt % in H_2_O), *N*-isopropylacrylamide (NIPAAm, 97%), *N*,*N*’-methylenebisacrylamide cross-linker (BIS, 99%), and 2-oxoglutaric acid (2-oxo, 99.0–101.0%) were obtained from MilliporeSigma. Deionized (DI) water (18 MΩ·cm, Cascada LS MK2, Pall) was used for hydrogel fabrication. 1/2′ × 1/2″ (thickness × width) multipurpose 6061 aluminum bars were purchased from McMaster Carr.

### Triple Network (TN) Hydrogel Fabrication

TN hydrogels were fabricated in a three-step UV cure process. Single network (SN) hydrogels were prepared and subsequently soaked in a double network (DN) precursor solution. Post soaking, hydrogels were removed from the solution and cured to form DN hydrogels. TN hydrogels were formed by performing a similar process after curing of the DN. The SN precursor solution consisted of AMPS (1.5 M), BIS cross-linker (4 mol % w.r.t. AMPS), and 2-oxo photoinitiator (0.1 mol % w.r.t. AMPS) in DI water. This solution was cured in a custom mold composed of glass slides separated by spacers (∼1 mm) on a UV plate (UVP Transilluminator PLUS, 6 mW cm^–2^, 365 nm, Analytik Jena) for 5 h, flipping every 15 min for the first hour and on the hour for the remaining 4 h to maintain symmetry. The cured SN hydrogels were then immersed in a DN precursor solution composed of NIPAAm (2.0 M), AAm (10 wt % w.r.t. NIPAAm), BIS (0.1 mol % w.r.t. NIPAAm), and 2-oxo (0.1 mol % w.r.t. NIPAAm) in DI water for 48 h. Post soaking, hydrogels were placed in a custom mold composed of glass slides separated by spacers (∼1.25 mm) and UV cured while immersed in an ice bath (∼7 °C) for 5 h following a similar flipping pattern to SN hydrogels. Cured DN hydrogels were immersed in a TN precursor solution composed of monomer (AMPS or APTAC; 0.5–2.0 M), BIS (0.1 mol % w.r.t. monomer), and 2-oxo (0.1 mol % w.r.t. monomer) in DI water for 48 h. After soaking, hydrogels were cured in a similar manner to DN hydrogels. Once cured, TN hydrogels were placed in DI water for at least 1 week before testing to reach equilibrium swelling. TN hydrogels were immediately tested upon removal from DI water to ensure minimal dehydration. TN hydrogels were denoted TN-*X*-*Y*M, where *X* represents the monomer (AMPS or APTAC) and *Y* represents the concentration (0.5–2.0 M; e.g., TN-AMPS-0.5M) of the 3^rd^ network. A DN hydrogel control (DN-AAm-10%) was fabricated similarly, but after curing the 2^nd^ network, the DN was lastly soaked in DI water without further modification.

### Interpenetrating Network (IPN) Hydrogel Fabrication

An interpenetrating network (IPN) hydrogel (IPN-AAm) was fabricated through a two-step, UV cure process in which SN hydrogels were soaked in a 2^nd^ network precursor solution and subsequently cured to form an IPN hydrogel. The SN precursor solution consisted of AMPS (1.5 M), BIS (1 mol % w.r.t. AMPS), and 2-oxo (0.1 mol % w.r.t. AMPS) in DI water. The precursor solution was injected between two glass slides separated by ∼ 1 mm thick spacers and exposed to UV light (UV transilluminator, 6 mW cm^–2^, 365 nm) for 5 h while being flipped at standard intervals to maintain symmetry (similar to TN hydrogel fabrication). The SN hydrogel was removed from the mold and immersed in the IPN precursor solution for 48 h. The IPN precursor solution consisted of AAm (1.5 M), BIS (0.1 mol % w.r.t. AAm), and 2-oxo (0.1 mol % w.r.t. AAm) in DI water. After soaking, the hydrogel was enclosed in a mold of two glass slides separated by spacers (∼1.25 mm) and then UV cured for 5 h flipping at the standard intervals. The resulting IPN hydrogels were then removed from the molds and soaked in DI water for 1 week before testing.

### Equilibrium Water Content (EWC)

The water content of the hydrogels was determined by comparing the weights of swollen and dried hydrogel discs. Hydrogel discs (6 mm × ∼2.5 mm, diameter × thickness) were punched out using a biopsy punch, and surface water was removed by blotting dry with a Kim Wipe (*n* ≥ 5). Hydrogels were then placed in an oven at 60 °C and dried overnight under vacuum (30 in. Hg). Water content was calculated as , where *W*_s_ is the swollen weight and *W*_d_ is the dry weight.

### Unconfined Compression

Compressive mechanical properties of hydrogels were determined using an Instron 5944 at room temperature. Hydrogel discs (6 mm × ∼2.5 mm, diameter × thickness) were punched out using a biopsy punch, and surface water was removed by blotting dry with a Kim Wipe (*n* ≥ 5). Hydrogel samples were preloaded with a force of 0.2 N, and the strain was zeroed. Samples were compressed at a displacement rate of 1 mm min^–1^ until fracture. Fracture was defined as a sharp drop in stress. The elastic modulus was defined as the slope of the linear region (0–10% strain) of the stress–strain curve. Strength was designated as the stress at fracture. Toughness was determined by the integration of the stress–strain curve to the point of fracture.

### Tension

Tensile mechanical properties (modulus, strength, toughness) of hydrogels were determined using an Instron 5944 at room temperature. Hydrogels were punched into dog bones using a certified punch (ASTM D1708-18) (*n* ≥ 5). Surface water was removed by blotting samples dry with a Kim Wipe. A preload of 0.2 N was applied to the samples to remove slack, and the strain was zeroed. Samples were displaced at a rate of 10 mm min^–1^. The elastic modulus was defined as the slope of the linear region (0–10% strain) of the stress–strain curve. High strains (∼100%) caused specimens to slip from clamps, preventing measurement of tensile strength, strain, and toughness.

### Lap Shear

For lap shear tests, hydrogels were adhered together using a custom mold to ensure consistent alignment (Figure S1a). Hydrogel samples were cut into 1 cm × 4 cm (width × length) strips using a cutting guide and razor blades. The strips were blotted dry to remove surface water and then placed in the custom mold, wherein the overlap of the strips formed a 1 cm^2^ connection (*n* ≥ 5). In the mold, pressure was applied by hand to the connection site for 1 min before adhered samples were removed. “Connected” hydrogels were soaked in DI water for 48 h before lap shear testing.

The interfacial shear strength of the connection was tested with an Instron 5944 at RT. Specimens were evaluated in a modified lap shear setup, where supports added to the upper and lower tension clamps prevented the rotation of the samples (Figure S1b).^56^ Supports were fabricated from 1/8 inch thick aluminum bars (McMaster Carr) cut to 0.5 in × 2.75 in (width × length). Each support had sandpaper attached to one side, to prevent displacement of the hydrogel. The supports were affixed along with hydrogel specimens in the tension clamps with 1 cm of the hydrogel in the clamp. Once inserted, a preload of 0.2 N was applied. Then, samples were displaced at a rate of 10 mm min^–1^, applying shear strain to the connection interface, until failure. Strength was defined as the stress at the point of failure of the interface or fracture of a hydrogel.

### Statistical Analysis

For unconfined compression, tension and EWC statistical analyses were completed using a two-way analysis of variance (ANOVA) with Dunnet’s multiple comparison test. For lap shear, statistical analyses were completed using one-way analysis of variance (ANOVA) with Dunnet’s multiple comparison test. All analyses were conducted with GraphPad Prism (Version 9.2.0) using a standard α level of 0.05. All comparisons with *p* < 0.05 were considered statistically significant.

## Results and Discussion

### TN Hydrogel Fabrication

TN hydrogels were fabricated in a three-step sequential UV cure process wherein after the 1^st^ and 2^nd^ cure, the resulting DN hydrogel was soaked in a precursor solution of the 3^rd^ network and then cured. The 1^st^ network was composed of tightly cross-linked, anionic PAMPS, and the 2^nd^ network was loosely cross-linked P(NIPAAm*-co*-AAm). The 3^rd^ network was formed from loosely cross-linking APTAC (cationic) or PAMPS (anionic) monomers of systematically tuned concentrations (0.5–2.0 M). Following the cure of the 3^rd^ network, the resulting TN-APTAC and TN-AMPS hydrogels were soaked in DI water for at least 1 week before testing. TN hydrogels were denoted TN-*X*-*Y*M, where *X* represent the 3^rd^ network monomer and *Y* represents the 3^rd^ network molar concentration (e.g., TN-AMPS-1.0M; Table 1). The DN hydrogel (DN-AAm-10%, i.e., formed after the 2^nd^ cure) that preceded the formation of TN hydrogels was included as a control.

**Table 1:** Hydrogel Network Compositions

	composition				
	1^st^ network^a^	2^nd^ network^b^		3^rd^ network^c^	
hydrogel	AMPS	NIPAAm	AAm (w.r.t. NIPAAm)	APTAC	AMPS
Single Network					
SN-AMPS-1.5M	1.5 M				
Double Network					
DN-AAM-10%	1.5 M	2.0 M	10 wt %		
Triple Network					
TN-APTAC-0.5M	1.5 M	2.0 M	10 wt %	0.5 M	
TN-APTAC-1.0M	1.5 M	2.0 M	10 wt %	1.0 M	
TN-APTAC-1.5M	1.5 M	2.0 M	10 wt %	1.5 M	
TN-APTAC-2.0M	1.5 M	2.0 M	10 wt %	2.0 M	
TN-AMPS-0.5M	1.5 M	2.0 M	10 wt %		0.5 M
TN-AMPS-1.0M	1.5 M	2.0 M	10 wt %		1.0 M
TN-AMPS-1.5M	1.5 M	2.0 M	10 wt %		1.5 M
TN-AMPS-2.0M	1.5 M	2.0 M	10 wt %		2.0 M

a 4 mol % BIS cross-linker w.r.t. AMPS, 0.1 mol % 2-oxo photoinitiator w.r.t. NIPAAm.

b 0.1 mol % BIS cross-linker w.r.t. NIPAAm, 0.1 mol % 2-oxo photoinitiator w.r.t. NIPAAm.

c 0.1 mol % BIS cross-linker w.r.t. monomer, 0.1 mol % 2-oxo photoinitiator w.r.t. monomer.

All TN hydrogels comprised an anionic 1^st^ network and a neutral 2^nd^ network, giving rise to intranetwork electrostatic repulsive and intranetwork hydrophobic interactions. However, resulting TN hydrogels varied in terms of internetwork cross-linking between the anionic 1^st^ network and the 3^rd^ network. These internetwork cross-links were electrostatically attractive in nature for TN-APTAC hydrogels and electrostatically repulsive in the case of TN-AMPS hydrogels. It was observed that TN-AMPS hydrogels expanded more while soaking in DI water (Figure S2). This was attributed to electrostatic repulsion between the 1^st^ and 3^rd^ anionic networks.

### Equilibrium Water Content and Mechanical Properties

Prior to the assessment of adhesivity, TN hydrogels were individually assessed in terms of water content and mechanical properties. Given that the hydration of native cartilage tissues (60–90% water) gives rise to functional bulk mechanical properties as well as tribological properties, this should be ideally recapitulated in hydrogel cartilage substitutes. Both compressive and tensile moduli were assessed, as some cartilage tissues can undergo loading in tension.^6,10,11,57^ Moduli were assessed at physiologically relevant strains (<10%) to avoid inflation due to strain hardening effects. In compression, strength, as well as strain at break and toughness, was also measured. However, under tension, high strains (∼100%) caused specimens to slip from clamps and thus prohibited the measurement of tensile strength and toughness.

**Figure 3:**
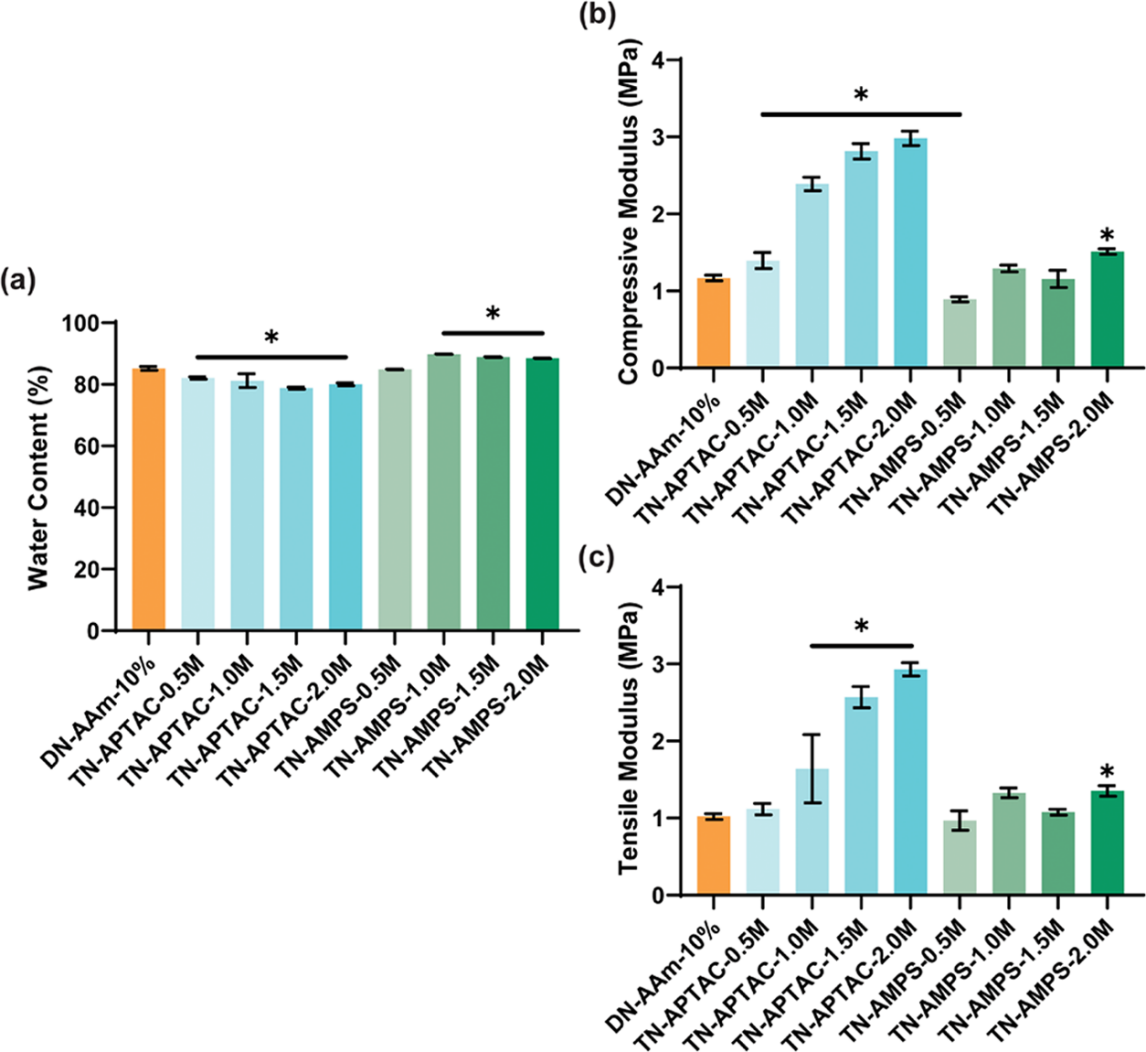
Material properties of electrostatic TN hydrogels: (a) equilibrium water content (EWC), (b) compressive modulus, and (c) tensile modulus. *Denotes the statistical difference (*p* < 0.05) from DN-AAm-10%.^53^

As previously reported, TN-APTAC hydrogels achieved an unprecedented combination of high hydration (∼80%) as well as ultrahigh moduli (∼1 to ∼3.0 MPa) and high compressive strengths (∼23 to 32 MPa; Figures 3 and S3 and Tables S1–S3).^53^ The increase in moduli with greater APTAC concentration in the 3^rd^ network was attributed to the concomitant increase in internetwork cross-links arising from electrostatic attractive forces. The dynamic nature of intranetwork and internetwork cross-links allowed TN-APTAC hydrogels to undergo appreciable compressive strains before failure (∼90%) and to achieve high toughness values (∼4 MJ/m^3^). TN-AMPS hydrogels obtained somewhat higher water contents (∼90%), thought to stem from the electrostatic repulsion between the 1^st^ and 3^rd^ networks (Figure 3 and Table S1). The moduli of the TN-AMPS hydrogels were generally lower (∼1.0 to ∼1.5 MPa) relative to the TN-APTAC hydrogels and were similar to DN-AAm-10% (∼1.2 MPa;^3^ and Tables S2 and S3). This reduction in modulus may be attributed in part to greater swelling. Still, the moduli of TN-AMPS hydrogels remain within the range of certain cartilage tissues (e.g., articular cartilage) and were much higher than conventional hydrogels such as PEG-diacrylate (PEG-DA; *E*_compressive_ ∼ 200 kPa and *E*_tensile_ ∼ 35 kPa).^50,58^ TN-AMPS hydrogels also exhibited relatively lower compressive strengths versus TN-APTAC hydrogels (Figure S3 and Table S2). While TN-AMPS-0.5M exhibited a high compressive strength (∼18 MPa) similar to DN-AAm-10%, a marked decrease was observed for TN-AMPS-1.0M, −1.5M, and −2.0M hydrogels (∼5 MPa). This coincided with a decrease in compressive strain (∼90 to ∼71%) and a decrease in toughness (∼3 to ∼1 MJ/m^3^). These results point to internetwork repulsive forces, giving rise to chain stiffening of networks and a subsequent inability to dissipate stress. Overall, TN-APTAC and TN-AMPS achieved cartilage-like hydration and moduli of several cartilage tissues.

### Adhesivity

**Figure 4:**
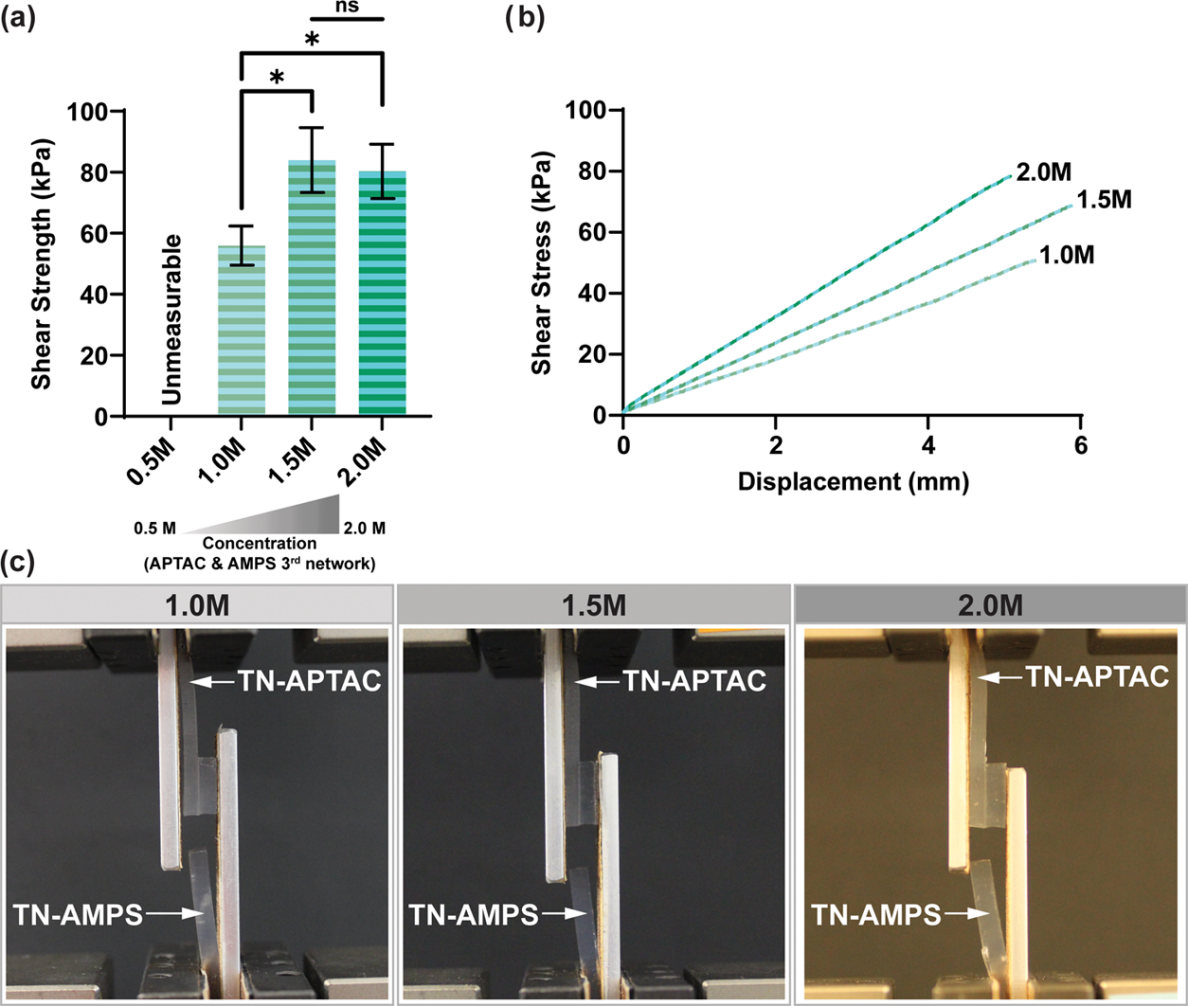
Using lap shear tests, TN-APTAC (cationic 3^rd^ network) and TN-AMPS (anionic 3^rd^ network) were evaluated for adhesion to one another when prepared with the same 3^rd^ network concentration (0.5, 1.0, 1.5, and 2.0 M): (a) shear strength of interface, (b) representative stress–displacement curves (note: shear strain not calculated as deformation cannot be solely attributed to adhered interface region), and (c) photographs showing cohesive failure occurred in TN-AMPS hydrogels in a tensile mode (i.e., perpendicular to the shear plane). *Denotes statistical difference (*p* < 0.05) versus TN hydrogels prepared with a 1.0 M 3^rd^ network.

Since the final network is known to dictate the electrostatic surface properties of multinetwork hydrogels,^54,55^ TN-APTAC and TN-AMPS hydrogels were expected to yield cationic and anionic surfaces, respectively. Such TN hydrogels of opposite charge have the potential to adhere to one another via electrostatic attractive forces. In the case of highly hydrated hydrogels, the effect of a dilute surface must be overcome by a sufficient concentration of moieties that overcome interactions with water and give rise to stable adhesion junctions.^47^ Thus, adhesivity was assessed between TN-APTAC and TN-AMPS hydrogels formed with 3^rd^ networks of the same concentrations (0.5–2.0 M). Interfacial shear strength was determined via lap shear tests wherein TN hydrogels were connected at a 10 mm × 10 mm interface and displaced axially (tension) until failure (Figure S1). TN hydrogels prepared with 3^rd^ networks of the lowest concentration (TN-AMPS-0.5M and TN-APTAC-0.5M) resulted in adhesive failure when a minimal (unmeasurable) force was applied (Figure 4 and Table S4). As the 3^rd^ network concentration was increased, interfacial adhesion improved, and cohesive failure was observed. TN-APTAC-1.0M and TN-AMPS-1.0M achieved a shear strength of ∼55 kPa. When the 3^rd^ network concentration was further increased to 1.5 and 2.0 M, shear strengths increased to ∼80 kPa. Thus, the higher concentrations of the 3^rd^ network indeed achieved the necessary concentration of electrostatic charge to form effective adhesion junctions. This increase in shear strength is attributed to increased charge density at the surfaces. Cohesive failure was consistently observed to occur in the TN-AMPS hydrogel due to lower ductility versus TN-APTAC hydrogels. Also, a tensile failure mode (i.e., perpendicular to the shear plane) was observed rather than the shear failure mode. For such scenarios when the failure occurs out of plane, the true “adhesion strength” can be expected to in fact be higher than what is measured.^48^ Overall, TN hydrogels formed with higher 3^rd^ network concentrations achieved the desired adhesivity by forming stable adhesion junctions based on electrostatic attractive forces at their surfaces.

### Adhesive TN Hydrogels to Build Cartilage-Like Constructs

**Figure 5:**
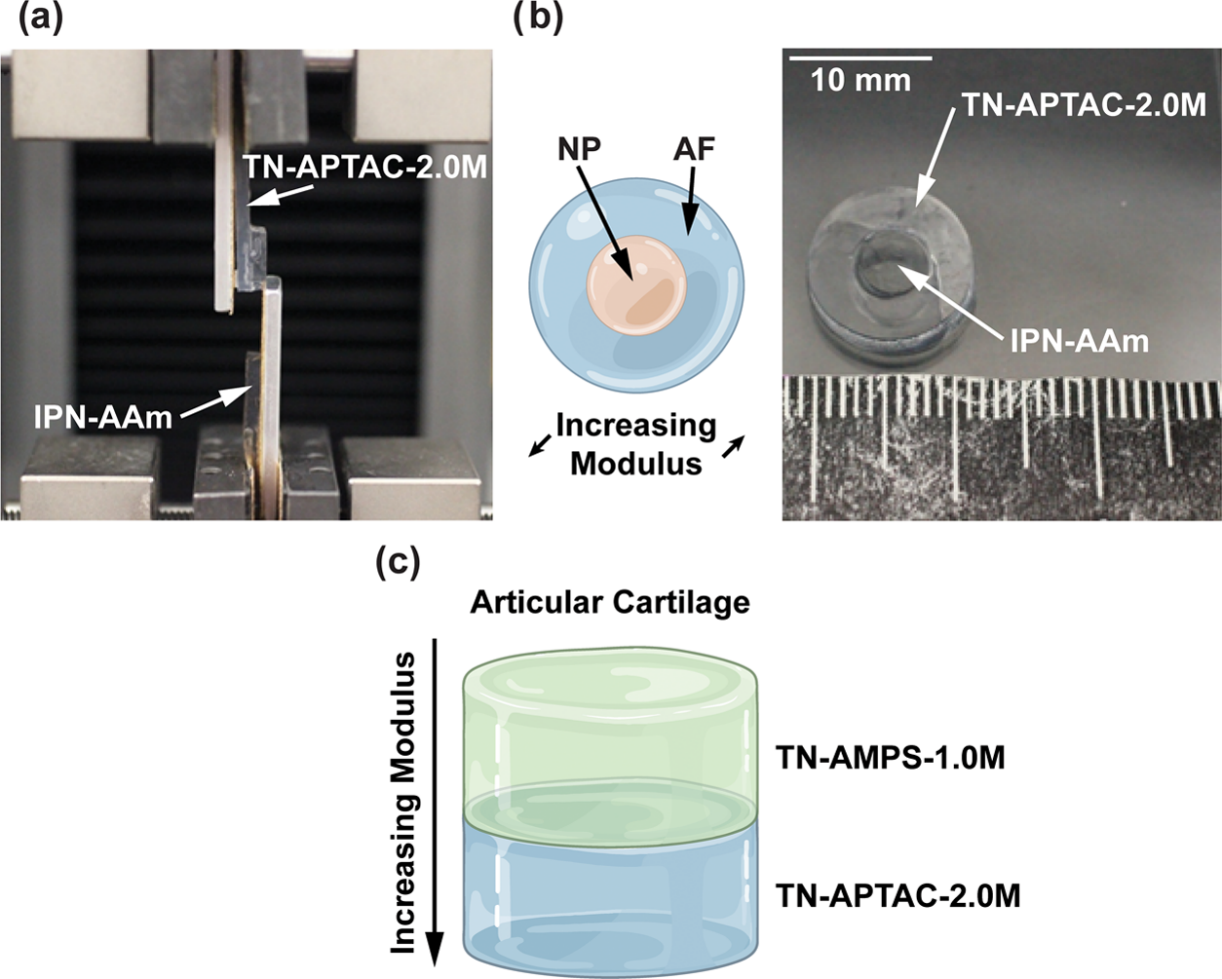
(a) Lap shear test of TN-APATAC-2.0M (cationic 3^rd^ network) [representing the AF of an IVD] and anionic IPN-AAm [representing the NP of an IVD], resulting in cohesive failure in IPN-AAm. (b) Schematic and photograph of the fabricated “IVD-like” construct. (c) Schematic of the proposed design of adhered TN hydrogels for the development of an articular cartilage replacement with regional (depthwise) moduli differences.

Such TN hydrogels have the potential to prepare cartilage constructs with regional properties in radial or depthwise directions (Figure 1). To demonstrate such utility, a proof-of-concept artificial IVD-like construct was formed. Two hydrogels were utilized to represent the AF and NP regions of an IVD. The more rigid AF region was represented with TN-APTAC-2.0M owing to its similar compressive moduli (∼3 MPa). The gelatinous NP component was based on an anionic interpenetrating network (IPN) hydrogel composed of AMPS and AAm (IPN-AAm) and displayed targeted, high water content (∼97%) and a low compressive modulus (∼140 kPa; Tables S1 and S2) like that reported for native NP tissue.^3,14^ First, the adhesivity of TN-APTAC-2.0M and IPN-AAm was elucidated with lap shear testing. A shear stress of ∼13 kPa was reached before cohesive failure was observed, wherein IPN-AAm was fractured in a tensile failure mode (Figure 5a and Table S4). To form the artificial IVD construct, a 12 mm diameter biopsy punch was used to create a disc of TN-APTAC-2.0M (∼2.5 mm thick). A center hole (5 mm diameter) was punched out of the disc. Then, a 5 mm diameter disc of IPN-AAm was inserted into the hole (using a guide to avoid contact with TN-APTAC-2.0M and improper adhesion during insertion; Figure 5b). Further illustrating the utility of adhesive TN hydrogels, a bilayer construct, improving on monolithic designs (e.g., Cartiva—a poly(vinyl alcohol) hydrogel with FDA approval for the toe joint),^59^ for an articular cartilage-like construct was produced (Figure 5c). Here, a TN-AMPS-1.0M hydrogel (*E* ∼ 1 MPa) and a TN-APTAC-2.0M hydrogel (*E* ∼ 3 MPa) represented the superficial and deep layers, respectively.

## Conclusions

Cartilage substitutes must replicate the regionally dependent moduli of native cartilage tissues to achieve the necessary performance. This work reported electrostatically adhesive TN hydrogels that have cartilage-mimetic hydration as well as moduli and so are useful building blocks for the development of such substitutes. TN hydrogels were fabricated with anionic (PAMPS) or cationic (PAPTAC) 3^rd^ networks, thereby controlling the surface charge. This afforded the potential to form adhesive junctions via electrostatic attractive forces between oppositely charged hydrogel surfaces. Increasing concentration of the 3^rd^ network likely led to improved adhesivity, attributed to greater charge density on the hydrated surface. Correspondingly, excellent adhesion was achieved as exemplified by cohesive failure, rather than adhesive failure at the interface. The utility of adhesive, TN hydrogels to form cartilage constructs with regional differences in the modulus was demonstrated. To form an IVD construct, a rigid TN-APTAC (cationic surface) hydrogel was connected to a gelatinous anionic hydrogel, representing the AF and NP, respectively. A conceptual design for articular cartilage further depicted the ability of these hydrogels to form heterogeneous synthetic cartilage replacements. Future studies of these TN hydrogels will focus on surface chemistry and charge characterization using scanning kelvin probe microscopy. Furthermore, the evaluation of their adhesive and mechanical properties in different solutions (e.g., phosphate-buffered saline or synovial fluid) will be carried out. Overall, this work establishes the realization of adhesive hydrogels with cartilage-mimetic mechanical and hydration properties and their ability to serve as a platform for the development of heterogenic synthetic cartilage replacements.

## Supporting Information Available

Swelling comparison figures; lap shear fixture diagram; and material property graphs and tables (e.g., mechanical properties, water content) (PDF)
