# Supplementary material for: Adhesive Hydrogel Building Blocks to Reconstruct Complex Cartilage Tissues
Source: ACS Biomater Sci Eng. 2023 Mar 7;9(4):1952–60. doi: 10.1021/acsbiomaterials.2c01438 (PMC10848198; doi:10.1021/acsbiomaterials.2c01438)
Supplement: Supplementary file 1 — ab2c01438_si_001.pdf [file ab2c01438_si_001.pdf]

SUPPORTING INFORMATION

Adhesive Hydrogel Building Blocks to Reconstruct Complex Cartilage Tissues

*Connor J. Demott[1], McKenzie R. Jones[1], Caleb D. Chesney[1], and Melissa A. Grunlan[1,2,3]**

[1]Department of Biomedical Engineering, Texas A&M University, College Station, TX 77843-3003 (USA)

[2]Department of Materials Science & Engineering, Texas A&M University, College Station, TX 77843-3003 (USA)

[3]Department of Chemistry, Texas A&M University, College Station, TX 77843-3003 (USA)

*Corresponding author email: mgrunlan@tamu.edu

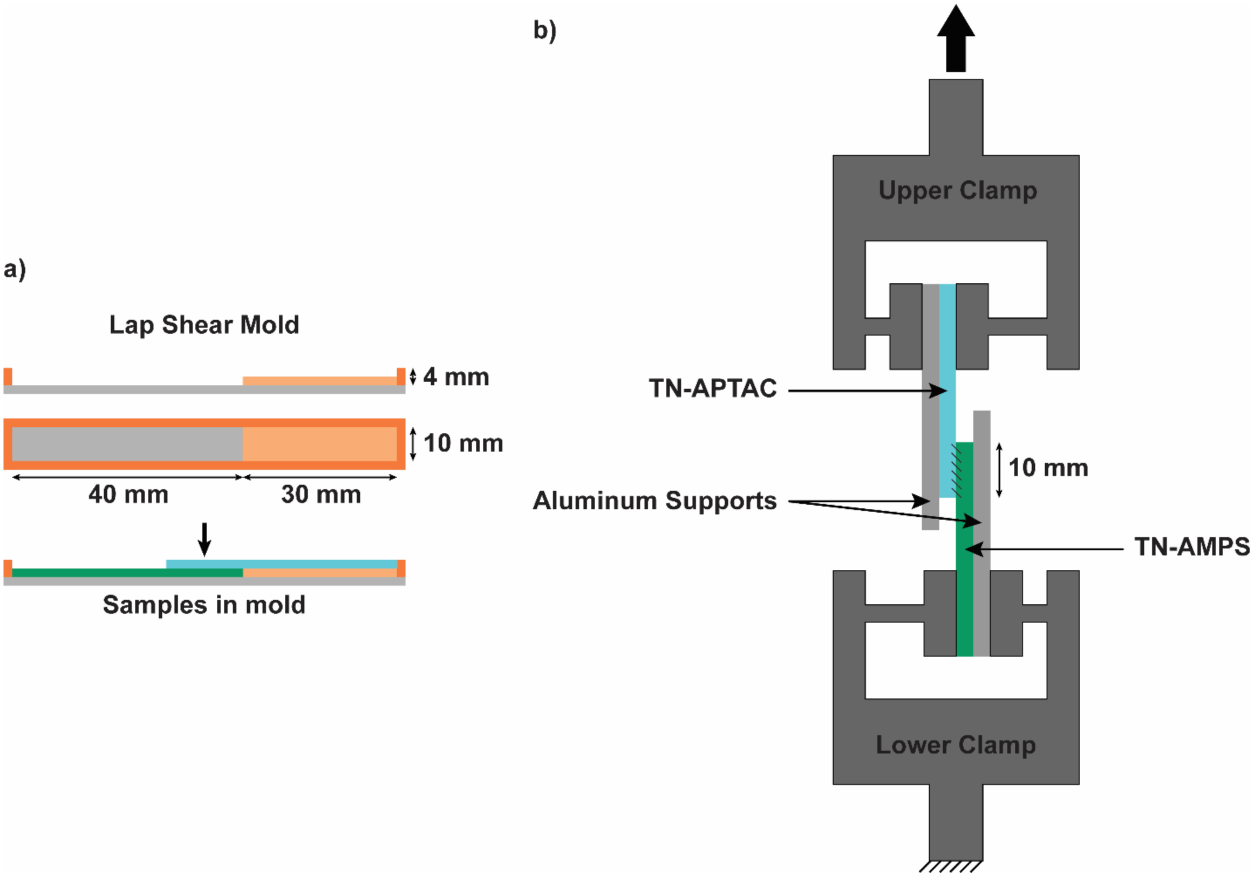

Figure S1. Lap shear test configuration: **(a)** mold to adhere hydrogel samples and **(b)** lap shear setup on Instron.

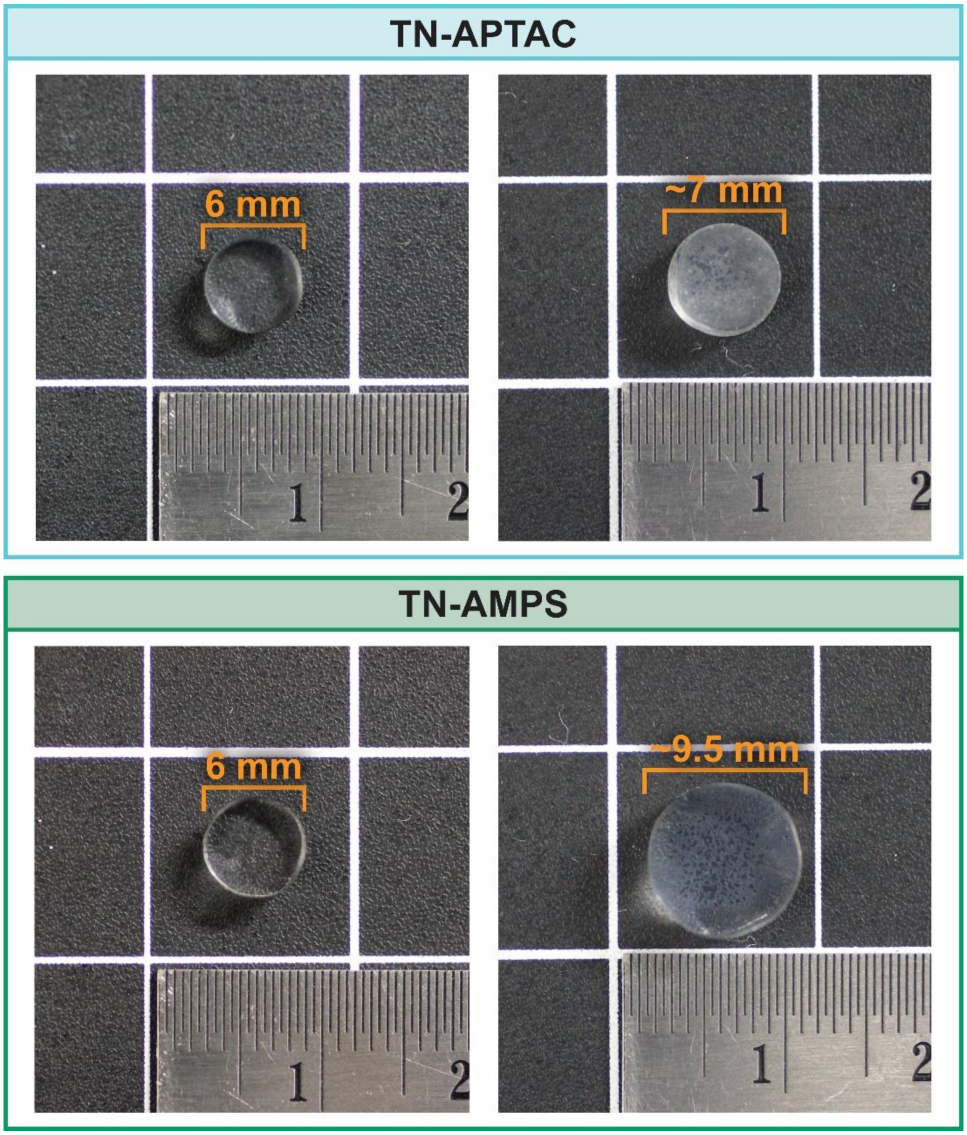

Figure S2. TN (1.5 M 3rd network) hydrogel swelling after cure of 3rd network and subsequent equilibration in DI water. **(LEFT)** Prior to equilibration in water, **(RIGHT)** after equilibration.

Table S1. TN hydrogel equilibrium water content (per **Figure 3a**). (Note: IPN-AAm also reported).[1]

Hydrogel	Water Content (%)
Double Network	
DN-AAm-10%	85.14 ± 0.61
Triple Network	
TN-APTAC-0.5M	82.09 ± 0.39
TN-APTAC-1.0M	81.19 ± 2.24
TN-APTAC-1.5M	78.79 ± 0.33
TN-APTAC-2.0M	80.01 ± 0.48
TN-AMPS-0.5M	84.84 ± 0.16
TN-AMPS-1.0M	89.79 ± 0.11
TN-AMPS-1.5M	88.83 ± 0.13
TN-AMPS-2.0M	88.45 ± 0.07
Interpenetrating Network	
IPN-AAm	96.50 ± 0.74

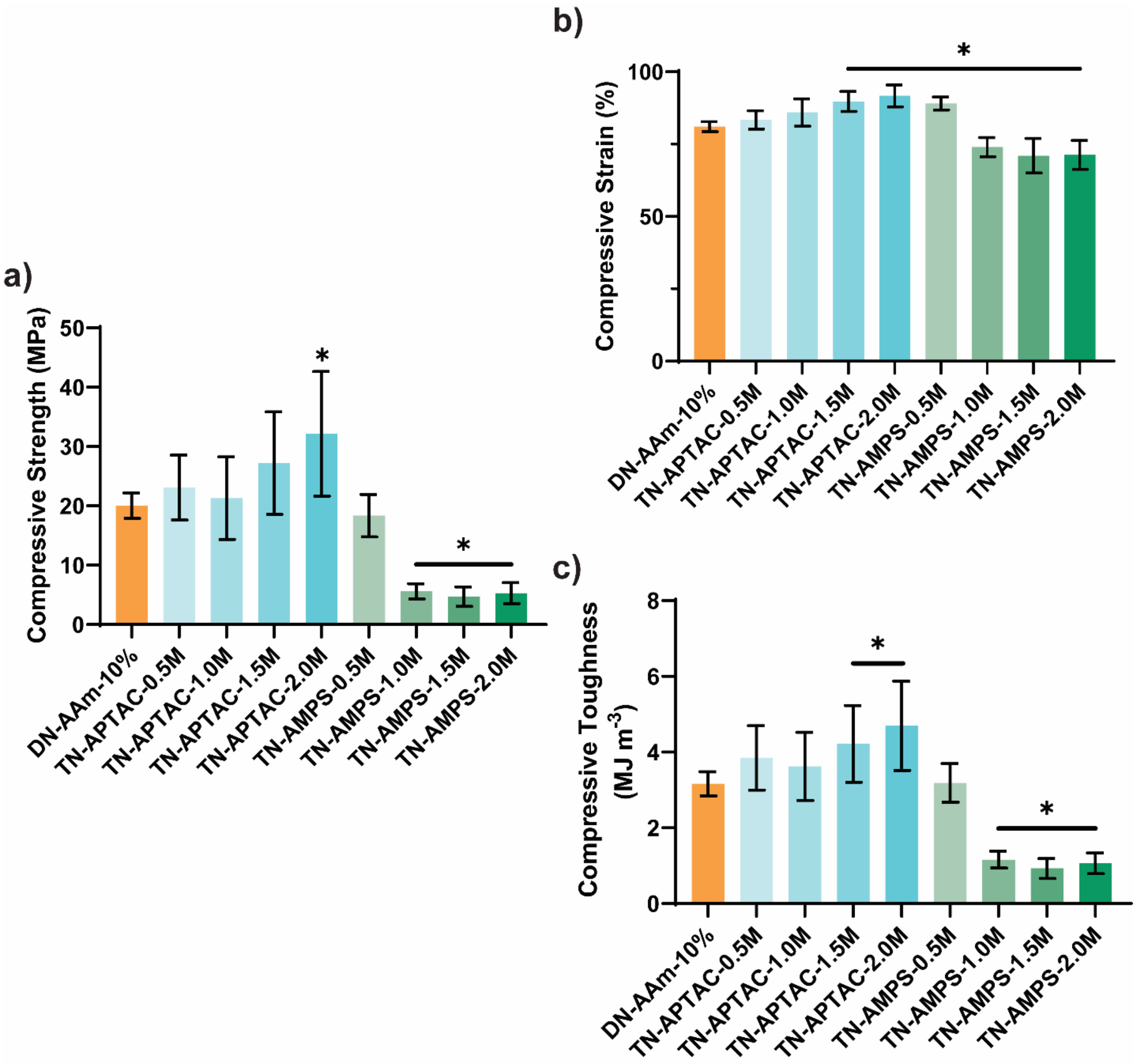

Figure S3. Compressive properties of TN hydrogels: **(a)** strength **(b)** strain at break, and **(c)** toughness. *denotes statistical difference ($p < 0.05$) from DN-AAm-10%.[1]

Table S2. TN hydrogel compressive mechanical properties (per **Figures 3** and **S3**). (Note: IPN-AAm also reported).[1]

Hydrogel	Modulus (MPa) (1-10% ε)	Strength (MPa)	Ultimate Strain (%)	Toughness (MJ m^{-3})
Double Network				
DN-AAm-10%	1.17 ± 0.04	20.04 ± 2.15	81.06 ± 1.77	3.16 ± 0.32
Triple Network				
TN-APTAC-0.5M	1.40 ± 0.10	23.09 ± 5.47	83.35 ± 3.17	3.85 ± 0.85
TN-APTAC-1.0M	2.39 ± 0.09	21.32 ± 6.97	85.93 ± 4.68	3.62 ± 0.90
TN-APTAC-1.5M	2.82 ± 0.10	27.20 ± 8.65	89.72 ± 3.47	4.22 ± 1.01
TN-APTAC-2.0M	2.98 ± 0.09	32.15 ± 10.50	91.69 ± 3.79	4.70 ± 1.18
TN-AMPS-0.5M	0.89 ± 0.03	18.34 ± 3.59	89.05 ± 2.22	3.19 ± 0.52
TN-AMPS-1.0M	1.29 ± 0.04	5.59 ± 1.29	73.96 ± 3.33	1.16 ± 0.22
TN-AMPS-1.5M	1.16 ± 0.11	4.71 ± 1.63	71.00 ± 5.92	0.93 ± 0.27
TN-AMPS-2.0M	1.51 ± 0.04	5.29 ± 1.79	71.28 ± 5.01	1.07 ± 0.28
Interpenetrating Network				
IPN-AAm	0.14 ± 0.00	1.77 ± 0.76	74.91 ± 7.28	0.31 ± 0.13

Table S3. TN hydrogel tensile moduli (per **Figure 3**).[1]

Hydrogel	Modulus (MPa)
Double Network	
DN-AAm-10%	1.02 ± 0.04
Triple Network	
TN-APTAC-0.5M	1.12 ± 0.08
TN-APTAC-1.0M	1.64 ± 0.44
TN-APTAC-1.5M	2.57 ± 0.14
TN-APTAC-2.0M	2.93 ± 0.09
TN-AMPS-0.5M	0.97 ± 0.13
TN-AMPS-1.0M	1.33 ± 0.06
TN-AMPS-1.5M	1.08 ± 0.04
TN-AMPS-2.0M	1.35 ± 0.07

Table S4. Lap shear strengths (per **Figure 4**). (Note: TN-APTAC-2.0M w/ IPN-AAm included.).

Interface	Interfacial Shear Strength (kPa)
TN-AMPS w/ TN-APTAC: Concentration of 3rd network:	
0.5 M	Unmeasurable*
1.0 M	55.94 ± 6.41**
1.5 M	83.99 ± 10.63**
2.0 M	80.27 ± 8.90**
TN-APTAC-2.0M w/ IPN-AAm	
-	13.24 ± 0.99**

*Adhesive failure **Cohesive failure

REFERENCES

1. Demott, C. J.; Jones, M. R.; Chesney, C. D.; Yeisley, D. J.; Culibrk, R. A.; Hahn, M. S.; Grunlan, M. A., Ultra-high modulus hydrogels mimicking cartilage of the human body. *Macromol. Biosci.* **2022**, 2200283.
